# Hospital admissions for severe mental illness in England: Changes in equity of utilisation at the small area level between 2006 and 2010

**DOI:** 10.1016/j.socscimed.2014.09.036

**Published:** 2014-11

**Authors:** Jonathan White, Nils Gutacker, Rowena Jacobs, Anne Mason

**Affiliations:** Centre for Health Economics, University of York, York YO10 5DD, United Kingdom

**Keywords:** England, Healthcare disparities/trends, Hospitals/utilization, Mental health services, Regression analysis, Small-area analysis, Socioeconomic factors, State Medicine/Organization & Administration

## Abstract

Severe Mental Illness (SMI) encompasses a range of chronic conditions including schizophrenia, bipolar disorder and psychoses. Patients with SMI often require inpatient psychiatric care. Despite equity being a key objective in the English National Health Service (NHS) and in many other health care systems worldwide, little is known about the socio-economic equity of hospital care utilisation for patients with SMI and how it has changed over time. This analysis seeks to address that gap in the evidence base.

We exploit a five-year (2006–2010) panel dataset of admission rates at small area level (*n* = 162,410). The choice of control variables was informed by a systematic literature search. To assess changes in socio-economic equity of utilisation, OLS-based standardisation was first used to conduct analysis of discrete deprivation groups. Geographical inequity was then illustrated by plotting standardised and crude admission rates at local purchaser level. Lastly, formal statistical tests for changes in socio-economic equity of utilisation were applied to a continuous measure of deprivation using pooled negative binomial regression analysis, adjusting for a range of risk factors.

Our results suggest that one additional percentage point of area income deprivation is associated with a 1.5% (*p* < 0.001) increase in admissions for SMI after controlling for population size, age, sex, prevalence of SMI in the local population, as well as other need and supply factors. This finding is robust to sensitivity analyses, suggesting that a pro-poor inequality in utilisation exists for SMI-related inpatient services. One possible explanation is that the supply or quality of primary, community or social care for people with mental health problems is suboptimal in deprived areas. Although there is some evidence that inequity has reduced over time, the changes are small and not always robust to sensitivity analyses.

## Highlights

• High bed occupancy for Severe Mental Illness (SMI) suggests limited access to care.
• We analyse trends in equity of inpatient use within and across small areas.
• Those living in deprived areas were significantly more likely to use inpatient care.
• Changes in this inequality between 2006 and 2010 were small and not always significant.
• SMI admission rates vary substantially between health care commissioning groups.

## 1 Introduction

Severe Mental Illness (SMI) encompasses a range of serious and chronic conditions including schizophrenia, bipolar disorder and psychoses. Although they represent only a subset of all mental and behavioural disorders – lifetime schizophrenia prevalence rates for the Western world are estimated to be between .4% and 1.4% (Cannon and Jones, 1996), and bipolar disorder rates for several European countries are estimated to range from .1% to 2.4% (NCCMH, 2006) – these conditions are associated with substantial morbidity and mortality. Evidence from the case register of a large metropolitan mental health provider in England suggests that the life expectancy of SMI patients is 10–15 years lower than the national average (Chang et al., 2011), whereas data from Denmark, Finland and Sweden suggest the figure could be as high as 15–20 years (Wahlbeck et al., 2011). A recent global morbidity study attributed 3.5% of total Years Lost to Disability to schizophrenia and bipolar disorder combined (Vos et al., 2012). The two diseases alone are estimated to constitute 1.5% of the total Disability Adjusted Life Year burden of disease for the UK in 2010 (Murray et al., 2013) and 1.1% in 21 regions worldwide (Murray et al., 2012). Defined more broadly, mental illness is associated with substantial societal costs such as lost productivity and increased costs of treating co-morbid physical illness (Centre for Economic Performance, 2012).

Despite the impact of these conditions and the importance of hospital care in treating the most vulnerable patients, little is known about the socio-economic equity of inpatient psychiatric care utilisation for SMI and how it has changed over time. Recent evidence demonstrates increased demand yet significantly reduced supply of psychiatric inpatient beds in the English National Health Service (NHS). Between 2003/4 and 2011/12, the number of mental health detentions has increased by 6%, but the number of mental health beds fell by 31% (Health Service Journal, 2013). This is reflected in very high bed occupancy rates, as found by a review by the English health and care regulator (CQC, 2013) and by an independent review of access to acute and crisis mental health care (Mind, 2011). There is some evidence that this indicates insufficient access to care rather than an efficient match of beds to demand; examples include early discharges resulting in readmissions, children being admitted to adult psychiatric wards, higher admission severity thresholds, and a more-than-doubling in the number of patients receiving out-of-area emergency treatment (BBC News, 2013, BBC News, 2014). If resources are under pressure, this raises the question of which socio-economic groups and geographical areas receive sufficient service provision given their level of need.

Equity is a concern in many health care systems. In England, the NHS Constitution describes ‘a wider social duty to promote equality’ – with particular attention to sections of society where improvements in health and life expectancy are not keeping pace with the rest of the population – and states that ‘access to NHS services is based on clinical need, not an individual's ability to pay’ (Department of Health (2009)). Analysis of socio-economic equity can assess whether these objectives are achieved. This paper specifically considers the socio-economic equity of SMI hospital admissions and how it has changed in the English NHS between 2006 and 2010. Socio-economic equity is defined as equality of utilisation between different deprivation groups after having adjusted for need (Wagstaff et al., 1991). Geographical equity at the Clinical Commissioning Group (i.e. local purchasers of health care) level is also explored, given government policy focus on the reduction of ‘unwarranted’ regional variation (Department of Health (2013)), and because income inequality may also operate at regional level (Fone et al., 2013).

This paper makes a distinct contribution to the evidence base on equity of inpatient care utilisation in three ways: its focus on serious mental illness; examining temporal changes in equity; and the use of small area data on need (specifically disease prevalence). It is important to recognise that differences in SMI admission (after standardising for need) could be driven by variation in the supply and quality of primary or community care rather than by inequitable provision of hospital care. However, recent research has found a positive and significant relationship between primary care quality (as measured by indicators from the Quality and Outcomes Framework) and hospital admissions for SMI (Jacobs et al., Forthcoming).

### 1.1 Previous literature on equity of secondary care utilisation

While there has been substantial policy interest in equity of access to care in the English NHS and elsewhere, the academic contribution to the debate is rarely focused on SMI or on changes in equity of utilisation. We systematically searched economics and medical bibliographic databases to identify relevant literature. This identified 49 unique records. Titles were screened by two independent reviewers, and abstracts were then checked for relevance. A wide range of different empirical approaches are applied in the literature. These include Kakwani indices (Wagstaff et al., 1991), as used to assess the impact of a large increase in coronary revascularisation on socio-economic and gender equity in Finland (Hetemaa et al., 2003), multilevel Poisson regression as used in an English study of geographical and socio-demographic equity in total joint replacement of the hip and knee (Judge et al., 2010), and proportional hazards models. A review of the extent to which published research has been able to identify socio-economic inequities of access within the NHS (Goddard and Smith, 2001) highlights that many articles nonetheless focus on equity of utilisation (realised access) rather than the broader but less observable components of access, including availability, quality, out-of-pocket costs and provision of information.

Only two papers have specifically investigated changes in equity of secondary care utilisation (Cookson et al., 2007, Cookson et al., 2012). This is despite the more reasonable assumptions required to estimate changes rather than levels of socio-economic equity – it need only be assumed that trends in unobserved need move in parallel for different deprivation subgroups – as well as the relevance of measuring changes to assess policy impacts. The earlier article considers change in socio-economic inequality of elective hip replacement between 1991 and 2001 using small area data. The authors first calculated indirectly age-sex Standardised Utilisation Ratios (SURs) for each small area, and for deprivation quintiles of small areas. SURs are calculated as the number of observed admissions divided by the number of age-sex expected admissions. To quantify the extent of inequity, they then calculated a rate ratio of SURs between the most and least deprived quintiles, and a concentration index of deprivation-related inequality in the SURs between small areas. Concentration indices are derived from concentration curves, which plot the cumulative fraction of the population on the horizontal axis, ranked in ascending order of socio-economic status, with the cumulative fraction of utilisation on the vertical axis. The concentration index is then equal to twice the area between the curve and a 45-degree line representing perfect equality. Whilst the study avoided selection bias due to the whole-population coverage of its data, it did not standardise for need (other than through age and sex).

The second paper (Cookson et al., 2012) used an expanded dataset with a vector of population, supply and need variables (including disease prevalence collected as part of the English pay-for-performance scheme, the Quality and Outcomes Framework) in an 8-year panel. It applied a revised methodology to identify change in socio-economic equity of several different categories of health care utilisation over time, including inpatient admissions and outpatient visits. The authors first calculated SURs for discrete deprivation groups using OLS-based indirect standardisation for population, age, sex and disease prevalence. For each year, the number of admissions was regressed on population, age, sex, disease prevalence, deprivation and supply indicators. Need expected admissions were calculated as the predicted value with the deprivation and supply factors fixed at their mean value for that year, and SURs were then calculated as before. The results were presented graphically as deprivation gradients, with deprivation groups plotted on the horizontal axis in increasing order of deprivation, and SURs plotted on the vertical axis. Concentration curves or concentration indices were not computed because they are a composite of several possible trends over time. The remaining part of the paper applied a continuous measure of deprivation in a pooled negative binomial analysis to test for changes in equity of utilisation.

### 1.2 Review of the literature on the factors associated with hospital admission for severe mental illness

Although the methodology used by Cookson et al. (2012) is appropriate for a mental health context, their set of explanatory variables may not be. The Cookson paper is focused on high-level activity measures and admission rates for diseases that affect mostly older patients; SMI admissions may have very different determinants. A second literature review was therefore conducted to identify relevant variables for subsequent analysis, using a similar review methodology.

Alongside age and gender, commonly identified risk factors for SMI hospital admission included medication non-adherence (10 articles; positively related), ethnicity (5 articles; mixed directions of effect), living alone (2 articles; different directions of effect), rurality (3 articles; mixed directions of effect), medication type (13 articles; mixed directions of effect depending on the drugs being compared), psychiatric bed supply (2 articles; positively related) and disease severity (4 articles; positively related). Comorbid substance misuse was also a commonly cited risk factor, including both alcohol abuse (5 articles) and drug abuse (11 articles). Several articles considered the beneficial effect of social support, such as social network size, social fragmentation and family support. Ten articles found previous hospitalisations to be predictive of future hospitalisations. Further detail is provided in the supplementary data published alongside this article.

The core variables chosen for this analysis comprised population size, age and sex; the need variables included SMI prevalence, ethnicity, the percentage of residents living alone and the percentage of residents who are married, capturing social support and the extent of social networks; and the supply variables captured measures of primary, secondary, community and informal care supply. Variables for education and unemployment were excluded due to their likely collinearity with the deprivation variable of interest. Many of the commonly identified factors were captured, although some are an imperfect match for those identified in the literature review. Other factors were omitted entirely due to limited data availability, including alcohol and drug abuse, some measures of severity, and the supply and adherence of particular medications. As we are measuring changes in equity, however, we need only assume that these unobserved factors move in parallel for different deprivation subgroups over time.

## 2 Data

We extracted data on all hospital admissions with a primary diagnosis of psychosis (ICD-10: F20–F29) or bipolar disorder (ICD-10: F30–F31) during the period April 2006 to March 2011 from the Hospital Episode Statistics (HES) data warehouse. HES captures all publicly-funded inpatient activity in England and provides detailed information about clinical and socio-demographic characteristics of the patient. For the purpose of this analysis, inpatient stays were defined on the basis of finished continuous inpatient spells, which accounts for transfers between providers. The sample was defined as follows: non-maternity admissions for people aged 15 and over who were discharged before the end of our study period (31st March 2011) and have valid information on their small area of residence.

A small number of specialist mental health hospitals frequently used the ICD-10 code R69.X (“Unknown diagnosis”) as the primary diagnosis for patients who had been previously diagnosed with SMI. With the unrefined identification strategy described above, these admissions would be excluded from the dataset, resulting in implausibly small numbers of admissions in certain Clinical Commissioning Groups such as NHS South Tyneside (6 admissions between April 2010 and March 2011). Given that SMI is a chronic and enduring condition, the identification strategy was refined to include admissions with an unknown diagnosis if the patient had previously been hospitalised with a primary or secondary diagnosis of SMI – considering all hospital admissions since April 2001 – and if the consultant was contracted under a mental health speciality other than learning disabilities. We performed sensitivity analysis to explore the effect of excluding these cases from our admission estimates.

Admissions were aggregated to small areas (Lower Super Output Area (LSOA)), which formed the unit of analysis. The geography of 32,482 LSOAs was developed by the Office for National Statistics. Each LSOA has an average population of 1500 (range 1000 to 3000) and the boundaries were chosen to minimise the variation within each LSOA in terms of the tenure and accommodation type variables from the 2001 Census (Tait, 2012). As these variables are proxies for income and wealth, LSOAs are appropriate units of analysis when investigating deprivation. Using LSOAs as the unit of analysis offered a significantly higher level of detail than alternative geographies such as Primary Care Trusts (150 areas) or Local Authorities (152 or 326 areas depending on the definition chosen), whilst maintaining high data availability.

Deprivation at small area was measured through the income deprivation domain of the Economic Deprivation Index (EDI), which was produced by the Social Disadvantage Research Centre at the University of Oxford (McLennan et al., 2012). The data consist of the percentage of people aged below 60 that are living in households claiming either Income Support (IS) or income-based Jobseeker's Allowance (JSA-IB), which are both means-tested out-of-work benefits. The EDI was chosen because it is time-varying for the years 1999–2009 inclusive, is based on administrative data with high coverage, has a clear cardinal interpretation, has no health components that could create circularity, and its methodology is well established. The income domain was specifically chosen (rather than the employment domain or the overall EDI score) because the latter two measures are partly a function of the number of people claiming Incapacity Benefit or Severe Disablement Allowance. Mental health is cited in 44% of claims for these two health-related benefits (McInnes, 2012); the EDI employment domain and overall EDI score would therefore risk biasing estimated deprivation effects upwards if they were correlated with SMI prevalence. Whilst the EDI income domain is not related to mental health prevalence in this way, its use does carry some disadvantages. Firstly, it does not count people who are in work but have low or uncertain income. Secondly, no EDI data are available for 2010; income deprivation in 2010 is therefore assumed to have the same value as in 2009, which may fail to capture any deepening of the recession at that time. Thirdly, the EDI does not measure deprivation amongst pensioners, although they are less likely to be admitted to hospital for SMI (Chang et al., 2011). Lastly, numerators are censored if they are less than 10 in order to protect the identity of benefit recipients. For this analysis, rather than assume that such values (representing 1.4% of LSOA records for each year) are zero, they are replaced with a uniform random integer between 0 and 9.

We derived a range of further demand-side variables. For each LSOA, we recorded the SMI prevalence, ethnicity, the percentage who are married, and the percentage of single person households. SMI prevalence is available at GP practice level from the Quality and Outcomes Framework dataset; we attributed it to LSOAs on the basis of the number of patients in the practice residing in each LSOA area, as indicated by the Attribution Data Set (ADS). For example, if 50% of a practice's patients are resident in a particular LSOA, 50% of its SMI patients were apportioned to that LSOA. We also derived the count of the population aged 15+, and a vector of percentages of the population aged 15+ in various age-sex groups. 5-year age bands were used for these groups from age 15 upwards, with wider bands of 65–74 and 75+ in the older age groups, resulting in a roughly equal proportion of the sample in each group. Information on age and gender distributions was derived from the ADS, whereas information on LSOA characteristics is provided from Census data by the Office for National Statistics.

Supply-side variables captured primary, secondary, community and informal care, and differences in the structure of health care in rural and urban areas. Accessibility of primary care was controlled for using a measure of GP density; time-varying counts of Full Time Equivalent GPs were obtained from the General Medical Services dataset and were attributed to LSOAs. Secondary care accessibility was measured using the straight line distance between the population-weighted centroid of each LSOA and the nearest acute and mental health providers, as computed by Pythagoras' theorem. Variation in the supply of community care (including Mental Health Teams) and other regional differences was captured by computing a binary variable for each Primary Care Trust (PCT). Different PCTs might offer different services, prioritise differently and spend in different ways. The PCT for each LSOA was identified using the NHS Postcode Database for 2011; PCT geographies were approximately stable over the study period. The remaining supply variables were taken from the 2001 Census and comprise (i) the percentage of the population providing informal care, and (ii) indicator variables for towns, villages, and urban areas.

Descriptive statistics for the base case sample are presented in Table 1.

**Table 1:** Descriptive statistics for variables used in the base case dataset.

Variable name	Mean	SD	Min	Max
*Dependent variable (base case)*				
Count of admissions (base case)	1.17	1.80	.00	51.00
*Deprivation variable*				
EDI income score	12.30	10.50	.00	76.70
*Core variables (base case)*				
Population aged 15+	1383.00	314.00	21.00	14,986.00
Percentage males aged 15–19	7.89	2.66	.00	60.80
Percentage males aged 20–24	7.67	3.80	.67	60.20
Percentage males aged 25–29	8.24	3.41	.00	32.90
Percentage males aged 30–34	8.47	3.44	.76	39.40
Percentage males aged 35–39	9.55	2.57	.88	32.30
Percentage males aged 40–44	9.90	1.92	.00	24.30
Percentage males aged 45–49	9.05	1.70	.00	25.00
Percentage males aged 50–54	7.78	1.61	.00	16.30
Percentage males aged 55–59	7.47	1.97	.00	16.90
Percentage males aged 60–64	7.02	2.41	.00	17.70
Percentage males aged 65–74	9.69	3.62	.00	31.80
Percentage males aged 75+	7.27	3.54	.00	46.80
Percentage females aged 15–19	7.43	2.60	.34	54.80
Percentage females aged 20–24	7.79	4.93	.77	77.40
Percentage females aged 25–29	8.31	4.19	.00	38.10
Percentage females aged 30–34	8.04	3.28	.00	30.10
Percentage females aged 35–39	8.85	2.27	.00	23.50
Percentage females aged 40–44	9.12	1.97	.00	23.20
Percentage females aged 45–49	8.36	1.84	.00	37.50
Percentage females aged 50–54	7.28	1.73	.00	16.10
Percentage females aged 55–59	7.12	2.08	.00	17.70
Percentage females aged 60–64	6.82	2.42	.00	19.60
Percentage females aged 65–74	10.10	3.61	.00	34.10
Percentage females aged 75+	10.80	5.27	.00	51.20
*Need variables (base case)*				
SMI Prevalence per 1000 pop aged 15+	9.10	2.93	.00	116.00
Percentage white ethnicity	91.00	15.00	4.64	100.00
Percentage mixed ethnicity	1.31	1.30	.00	14.10
Percentage Asian ethnicity	4.51	10.60	.00	93.70
Percentage black ethnicity	2.31	5.74	.00	62.20
Percentage other ethnicity	.88	1.39	.00	36.20
Percentage living alone	29.30	9.41	.64	86.70
Percentage married	40.60	9.93	2.81	69.00
*Supply variables (base case)*				
=1, if Town	.09	.29	.00	1.00
=1, if Village	.09	.29	.00	1.00
Distance to acute provider (miles)	5.30	4.97	.00	60.10
Distance to MH provider (miles)	13.70	10.60	.02	75.30
GP Density per 1000 pop aged 15+	.76	.15	.00	3.48
Percentage providing informal care	9.93	2.12	1.51	19.60

Note: sample size 162,410.

## 3 Methods

Following Cookson et al. (2012), socio-economic equity in health care is defined as equality in utilisation between different deprivation groups after having adjusted for need. This is a form of horizontal equity, meaning equal utilisation for equal need. The analysis is divided into two stages. The first stage is designed to identify changes in equity over time for particular discrete deprivation groups and provides a geographical analysis. The first stage applies OLS-based indirect standardisation for need and supply-side factors, with the results aggregated into discrete deprivation groups, and then aggregated to Clinical Commissioning Groups to conduct geographical analysis of equity (see section 3.1). The second stage is designed to provide an overall measure of whether access is pro-rich or pro-poor and to test whether this measure has changed over time. As access is measured by hospital admissions, a pro-poor relationship does not necessarily imply that access to community or social care is better or worse. The second stage uses a continuous measure of deprivation within a pooled negative binomial model to provide a formal test of whether changes over time in the pro-poor or pro-rich direction are statistically significant, after accounting for a range of covariates (see section 3.2). The second stage complements the first stage by explaining the direction of equity changes over time that were identified in the first stage.

### 3.1 Variation in standardised utilisation across deprivation groups and geographies

Need-expected utilisation was computed at the LSOA level using regression-based indirect standardisation methods (O'Donnell et al., 2008). The following equation was estimated by OLS, separately for each year of the data:

(1) $$adm_{i}=\alpha +D_{i}\varphi +P_{i}\beta +A_{i}^{\prime }\gamma +M_{i}\omega +N_{i}^{\prime }\delta +S_{i}^{\prime }\theta +\varepsilon_{i}$$

where *i* indexes the LSOA; *adm* denotes the number of SMI admissions; *D* denotes the Economic Deprivation Index income score; *P* denotes a count of the population aged 15+; A denotes a vector of variables recording the percentage of the population in each age category (separately by sex, with the variables for each sex summing to 100); *M* denotes SMI prevalence; *N* denotes the remaining vector of need variables; *S* denotes a vector of supply variables; and *ε* is an independent and identically distributed error term. The reference categories in this regression were men aged 25–29, women aged 25–29 and the white ethnicity category.

The need-expected number of admissions in a given LSOA and year was calculated as.

(2) $$a\hat{dm_{i}}=\hat{\alpha }+\bar{D}\hat{\varphi }+P_{i}\hat{\beta }+A_{i}^{\prime }\hat{\gamma }+M_{i}\hat{\omega }+N_{i}^{\prime }\hat{\delta }+\bar{S}\hat{\prime \theta }$$

where the deprivation and supply variables were fixed at their national mean values for that year in order to sterilise their effect (O'Donnell et al., 2008). This isolated the effect of deprivation in the analysis and ensured that the effects of higher supply are not conflated with higher need. If the supply and deprivation factors are correlated with the other explanatory variables but were excluded from the regression, the coefficients on the remaining variables would indirectly capture the effects of deprivation and supply due to omitted variable bias. Separately, despite the fact that a count data model would better fit the highly skewed nature of the dependent variable, OLS was used in this part of the analysis because the predictions in a non-linear model would be affected by the values at which the supply variables are fixed (O'Donnell et al., 2008).

Four deprivation groups were used in the discrete analysis. LSOAs were grouped such that either (i) less than 10%, (ii) 10–20%, (iii) 20–30%, or (iv) over 30% of the population are defined by the EDI as being income deprived. These groups were chosen to focus on the most deprived areas; the percentage of LSOAs in each category is 55.0%, 24.0%, 12.8% and 8.2% respectively. Standardised Utilisation Ratios (SURs) were calculated for a particular deprivation group in each year by dividing the number of observed admissions by the number of need expected admissions. A SUR of less than one indicates that utilisation in that deprivation group is lower than would be expected given their level of need. This may indicate inadequate access to inpatient care (or good access to high quality primary, community or social care). Standardised Utilisation Rates were computed by multiplying the appropriate SUR by the national mean utilisation rate. All rates are expressed per 100,000 population aged 15 or above. Standardised utilisation rates and ratios were also calculated at Clinical Commissioning Group (CCG) level by dividing the sum of observed admissions in a given CCG by the number of need expected admissions in that CCG.

### 3.2 Testing changes in equity over time

In order to test for changes in equity of utilisation over time, we estimated a negative binomial regression model (with NB2 variance function, see (Cameron and Trivedi, 2010)) on the number of admissions in each LSOA, including the same list of indicators for local need, supply of care, and deprivation as in the geographic analysis. Data were pooled over years and year effects were introduced to allow for temporal changes in utilisation. These year effects were interacted with deprivation to isolate changes in equity over time. All standard errors were clustered by LSOA in order to account for the correlation of observations over time (Rogers, 1993). As a robustness check, we also estimated a panel data model with random intercepts for each LSOA.

The assumptions needed to identify *changes* in the association between income deprivation and SMI admissions are far less restrictive than those needed in the standardisation analysis (Cookson et al., 2012). Specifically, a single assumption is required: that unobserved need for SMI care (i.e. need that has not been revealed through accessing services) did not increase more rapidly amongst income deprived patients relative to other members of society. Parallel trends in utilisation data lend support to this assumption of parallel trends in need, as current utilisation may be a determinant of future need. Some other factors that could differentially affect unobserved need, such as changes in medical technology and changes in the socioeconomic determinants of health, are unlikely to affect our study due to its relatively short time period. However, we cannot rule out the possibility that other factors, such as differential impacts of the economic recession, may weaken this assumption.

Five sensitivity analyses were performed. First, we excluded all episodes with an ICD-10 primary diagnosis code of R69.X (“Unknown diagnosis”). As discussed in the Data section, a small number of providers record high proportions of their admissions as unknown diagnoses; this sensitivity analysis tested our decision to include such episodes if the individual has SMI and was treated under a psychiatric specialty. Second, we dropped admissions for patients aged 75+ from the base case dataset, as older patients with a primary diagnosis of R69.X may have received psychiatric care for dementia rather than SMI. Thirdly, we re-estimated all models using as the dependent variable the count of patients with at least one admission. This tested the sensitivity of the results to a small number of ‘outlier’ LSOAs where a small number of individuals have multiple admissions. The fourth and fifth sensitivity analyses were the same as the second and third, but also excluded all episodes with an unknown primary diagnosis code, as in the first sensitivity analysis.

## 4 Results

### 4.1 Variation in standardised utilisation across deprivation groups and geographies

**Fig. 1:**
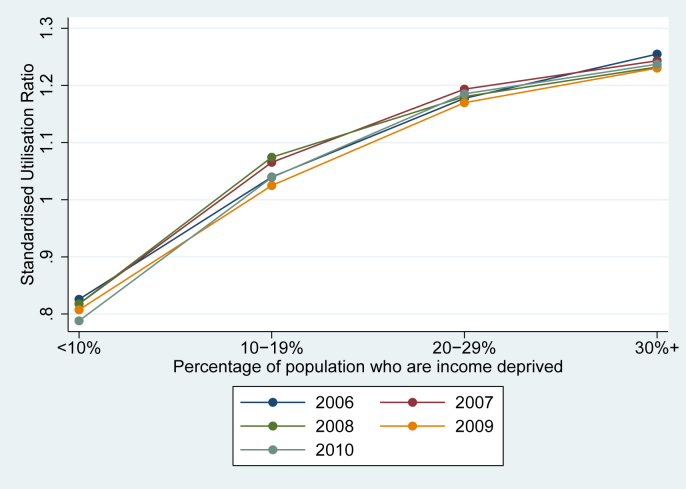
Estimated deprivation gradients for all years 2006–2010.

**Fig. 2:**
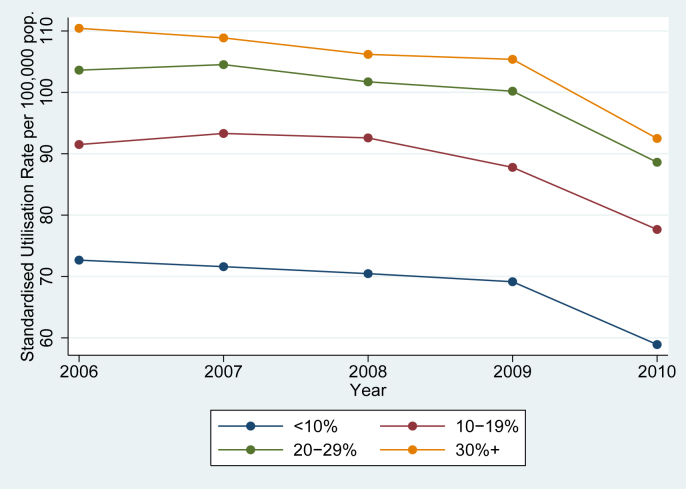
Trends in Standardised Utilisation Rates over time.

Fig. 1, Fig. 2 show the social gradient of SMI admission and how it has changed over time. Fig. 1 shows the SUR for each deprivation category in each of the five years. All lines are clearly upward-sloping, providing evidence that the equity of utilisation of SMI hospital care is pro-poor, and the relationships are remarkably consistent across years. (All but the lowest deprivation group have above-expected utilisation, and there is a concave relationship with each deprivation category associated with a smaller increase in utilisation. The appropriateness of this gradient depends on the social welfare function i.e. is a matter of the preferences of the stakeholders of the health care system; some might prefer a flat social gradient, although poorer patients are less likely to access private sector care. Given the wider social duty to promote equality that is enshrined in the NHS Constitution (see Introduction), a downward sloping social gradient would be cause for concern. Fig. 2 shows trends in the Standardised Utilisation Rate (rather than the ratio) by year and deprivation group, again showing a clear relationship between deprivation and standardised utilisation. The fall in utilisation in the last year of our sample is likely to be due to truncation, i.e. patients that had not finished their inpatient stay by the 31st of March 2011 and are therefore not recorded in our dataset. Trends are broadly parallel between groups, providing support for the assumption of constant relative need for SMI care across deprivation groups, i.e. need did not increase more rapidly for deprived patients compared with the rest of society.

### 4.2 Inequality of utilisation by geographic area

**Fig. 3:**
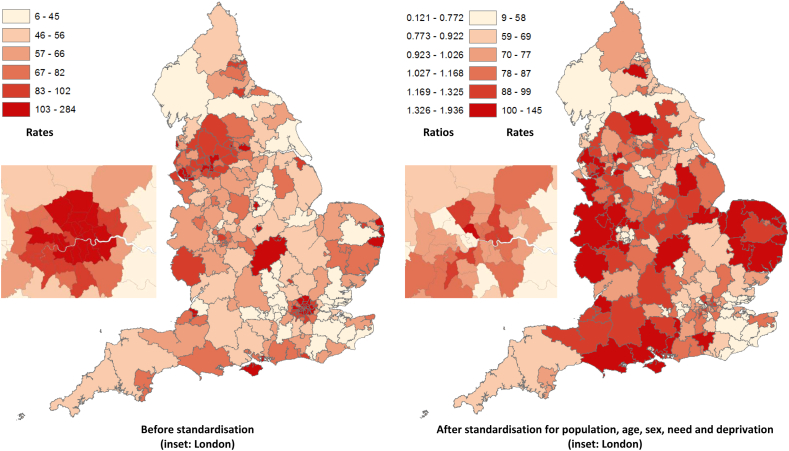
Clinical Commissioning Group-level maps showing the 2010/11 utilisation rate (crude and standardised) per 100,000 population aged 15 and above.

The reduction of unwarranted geographical inequality (often connected to the term ‘postcode lottery’) is part of the NHS Outcomes Framework which is used to hold the NHS to account (Department of Health (2013)). Fig. 3 presents an analysis of geographical (rather than socio-economic) equity at local purchaser (CCG) level. The left-hand map shows crude rates of SMI admission per 100,000 population. Darker areas (denoting high rates) include urban areas such as London, Bristol, Nottingham and Leicester, which tend to have younger populations, a larger share of ethnic minorities and a higher concentration of people in lower socioeconomic classifications, alongside Great Yarmouth and the Isle of Wight, which are far less densely populated and have a larger share of older people. The second map shows the impact of standardising for population, age, sex, need (including prevalence) and deprivation. The results can be interpreted either as Standardised Utilisation Rates or as Standardised Utilisation Ratios, with darker areas denoting above-expected utilisation. Importantly, in order to illustrate departures from the deprivation–utilisation relationship identified above, the effect of deprivation was not sterilised (held fixed) in these calculations. Deprived areas therefore have higher expected rates. Interestingly, some of the areas with high crude rates have below-expected standardised rates; in the top quartile of crude rates, 30% of CCGs have below-expected utilisation in 2010. This may imply that their high observed utilisation level is still insufficient to address the local level of need.

In the sensitivity analysis using a dependent variable based on admissions excluding those with unknown diagnosis (ICD-10: R69.X), findings are similar to the base case, demonstrating a clear, positive and consistent relationship between deprivation and standardised utilisation. However, there was a rise in standardised utilisation between 2006 and 2010 for the most deprived group. The sensitivity analysis of Fig. 3 (not shown but available on request) highlights the impact of removing R69-coded episodes in the unstandardized map for urban areas such as Newcastle and Bristol alongside Northamptonshire, although the maps are otherwise similar.

### 4.3 Changes in equity over time

Table 2 presents Incidence Rate Ratios (IRRs) for the key coefficients from the count regression models. The IRR indicates the proportional change in admissions associated with a one unit increase in the explanatory variable, so a variable is positively associated with SMI admissions if its IRR is greater than one. Many of the explanatory variables (including the EDI income score) are defined on a 0–100 scale so that a one unit increase can be conveniently interpreted as a one percentage-point increase.

**Table 2:** Summary of key coefficients in the base case, alternative case and sensitivity analyses.

Specification	Variables	Core model		Core/need model		Full model	
		IRR	SE	IRR	SE	IRR	SE
Base case HES identification strategy	EDI Income score	1.036***	.001	1.017***	.001	1.016***	.001
	Interaction Term	.998**	.001	.998+	.001	.998*	.001
Sensitivity analysis (1) (excluding admissions with primary diagnosis of R69)	EDI Income score	1.037***	.001	1.017***	.001	1.015***	.001
	Interaction Term	.999	.001	1.000	.001	.999	.001
Sensitivity analysis (2) (excluding patients aged 75+)	EDI Income score	1.037***	.001	1.016***	.001	1.016***	.001
	Interaction Term	.998**	.001	.998*	.001	.998*	.001
Sensitivity analysis (3) (count of those with >1 admission)	EDI Income score	1.035***	.001	1.015***	.001	1.014***	.001
	Interaction Term	.997***	.001	.998*	.001	.998*	.001
Sensitivity analysis (4) (like (1) but also excluding patients aged 75+)	EDI Income score	1.038***	.001	1.016***	.001	1.015***	.001
	Interaction Term	.999	.001	1.000	.001	.998+	.001
Sensitivity analysis (5) (like (1) & count of those with >1 admission)	EDI Income score	1.036***	.001	1.016***	.001	1.014***	.001
	Interaction Term	.998**	.001	.999	.001	.998*	.001

Notes: Interaction Term denotes an interaction between the EDI Income score and the dummy variable for the year 2010; IRR denotes Incidence Rate Ratio; SE denotes Standard Error; statistical significance is denoted as + (10%), * (5%), ** (1%) and *** (.1%).

The results indicate a highly statistically significant pro-poor relationship between income deprivation and hospital admissions for SMI in 2006. One additional percentage point in population income deprivation (according to the EDI definition) is associated with a 1.6% increase in SMI admissions. The IRRs for the interactions between income deprivation and year all have values around .999 or .998, suggesting that socio-economic equity of SMI admissions has become marginally less pro-poor. These changes are statistically significant (mostly at the 5% and 10% levels) in 2009 and 2010 (perhaps because the deprivation estimates are the same for both years). However, the small magnitude of the change suggests little policy relevance; the 1.6% impact of a percentage point increase in income deprivation on SMI admissions in 2006 (622 extra admissions nationally) becomes a 1.4% impact in 2010 (478 extra admissions). A model with random effects at the LSOA level yields very similar results.

The other explanatory variables in the full base case model are almost always statistically significant. Greater distances to the nearest mental health provider are positively and significantly associated with SMI admissions. This may be explained by the interaction of the health care provider with the population living in close proximity, allowing regular monitoring in an outpatient setting, and hence less inpatient admissions. The percentage of the population who are providing informal care is also positively associated with SMI admissions, and this may be because carers encourage engagement with health care services when appropriate.

The extent of the pro-poor relationship between deprivation and SMI admissions in 2006 is highly consistent (and significant at *p* < 0.001) across the sensitivity analyses reported in Table 2, although the association is notably stronger in models that only include the core explanatory variables. In the core/need and full models, an additional percentage point of EDI income deprivation is associated with around a 1.5% increase in SMI admissions. However, the interaction terms are not always statistically significant in the sensitivity analyses, so there is only tentative evidence of a small shift in the pro-rich direction between 2006 and 2010. In any case, the effect is small and of limited practical relevance.

## 5 Conclusions and discussion

Our analysis identifies inequalities in access to inpatient care for patients with severe mental illness using a five year panel of hospital admissions in the English NHS. Four important conclusions can be drawn from the findings of this study. Firstly, SMI admission is shown to be pro-poor for all years and a one percentage point increase in area income deprivation is consistently associated with a 1.5% proportionate increase in SMI admissions (*p* < 0.001 in all models). However, the lack of available data means that our analysis does not capture all the factors that the literature suggests are potentially associated with SMI admissions (e.g. alcohol and drug abuse, medication non-adherence and disease severity). If these factors cause deprivation, rather than merely be associated with deprivation, our analysis may overestimate the size of the association between deprivation and SMI admission. If these factors are also caused by deprivation, the relationship would be endogenous (i.e. interdependent). However, a lack of data on these factors means that we cannot resolve this issue. More broadly, a pro-poor relationship may not necessarily illustrate better overall access to care in more deprived areas; it could also indicate lower standards of primary, outpatient or informal care.

Secondly, the association between deprivation and SMI admission has remained broadly stable over our study period. A small change in the pro-rich direction is consistent across the base case and sensitivity analyses, although its statistical significance varies and the size of the effect is unlikely to be of policy relevance. This result is less vulnerable to the aforementioned partial coverage of need factors, as we need only assume that unobserved need for SMI care (i.e. need that has not been revealed through accessing services) did not increase in deprived groups relative to non-deprived groups between 2006 and 2010. This assumption is partially supported by the parallel movement of Standardised Utilisation Rates for different deprivation groups over time (Fig. 2); non-parallel trends in utilisation could otherwise result in non-parallel changes in future need. There is some evidence that deprivation does not predict schizophrenia incidence (Sariaslan et al., 2014), but the adverse health effects of the recession could nevertheless have been greater in more deprived areas. Other factors that could differentially affect unobserved need, such as changes in medical technology and changes in the socioeconomic determinants of health, are unlikely to affect our study due to its relatively short time period.

Thirdly, the geographical analysis identifies substantial regional variation in SMI admissions, even after controlling for population, age, sex, need (including prevalence) and deprivation. This is important due to government policy on the reduction of unwarranted geographical inequality and on ensuring parity of esteem between physical and mental health (Department of Health (2011)). Further investigation at a local level could distinguish between genuinely inadequate supply, high quality primary or community care and inadequate data quality. Commissioners of health care services would then be able to respond appropriately and ensure that access to publicly funded care for people with SMI is equitable.

Lastly, the analysis of Hospital Episode Statistics data highlights poor quality coding by certain providers. Primary diagnosis data are a crucial piece of clinical and management information that, despite the challenges of diagnostic coding in psychiatry, should be readily available. Mental health hospital providers are now being incentivised to code comprehensively through the Care Quality Commission regulatory standards so this may improve data quality in future.

A key strength of this study is that it uses admission data that cover the whole population (therefore avoiding the potential for selection bias in survey data) and data from GP practice information systems that provide information on area-level variation in prevalence rates for SMI. The main limitation of the study is its ecological nature; area-level data will not capture all variation in socio-economic deprivation at the individual level, although this is partly mitigated by the choice of the LSOA geography, which is small and has its boundaries set to minimise variation in terms of tenure and accommodation type. Other limitations are as follows. While we measure variation in access to secondary care due to income deprivation, we cannot determine whether this variation is brought about by unequal access to primary, secondary or community care, variation in the quality of preventive care in the primary and community care sectors, or whether it is driven by factors outside of the health care system. Separately, although a weaker assumption is needed to identify changes in need, we cannot rule out the possibility that other factors, such as the economic recession differentially affect those in more disadvantaged areas. Lastly, by focussing on patients treated within the NHS we may exclude the wealthiest patients who can afford to pay for their care privately. A substitution of wealthy private patients towards NHS care would be identified in our analysis as a worsening of equity. However, this effect is likely to be small as the vast majority of mental health hospital care in England is publicly funded. Specifically, the £143 m market for privately funded mental health hospital care (Laing and Buisson, 2013) compares with £2 billion of NHS spending on psychotic disorders (NHS England, 2014).

There are several avenues for further research. Firstly, to investigate the potentially interconnected role of alcohol and drug abuse, medication non-adherence, disease severity, and quality of preventative care alongside deprivation in determining SMI admission rates. Secondly, the 2006–2010 time period covered by this study only captures the impact of the early years of the recession, which may affect SMI patients though unemployment and through the financial impact on inpatient services. The closure of around 10% of mental health beds since April 2011 (BBC News, 2013) highlights the importance of extending this analysis of changes in equity of admission for further periods. Thirdly, further research could attempt to incorporate the number of private SMI admissions over the study period although such data is privately held and the proportion of private SMI patients in England is small (as discussed above). Fourthly, further research could control for spatial autocorrelation as a robustness check in area level equity analyses, thereby allowing for the possibility that SMI admissions in a given area are correlated with SMI admissions in surrounding areas. Lastly, the role of income inequality on admissions for severe mental illness, and how this interacts with economic deprivation, could also be investigated. Research on common mental illness in Wales found that the relationship between income inequality and economic deprivation was inter-related and complex (Fone et al., 2013).

In conclusion, hospital admission for SMI appears to be pro-poor, with only tentative evidence of a reduction in inequity between 2006 and 2010. Notable geographical inequity persists after controlling for population, age, sex, need and deprivation factors. It would be interesting to replicate this analysis in other countries with health systems that are either similar or dissimilar to the English NHS. This could help illuminate whether the pro-poor nature of hospital admissions for people with SMI is systemic, or whether other factors such as differences in the availability of substitute outpatient care, or more generally the funding and configuration of primary and community care, underlie our findings.
