# Supplementary material for: Hospital admissions for severe mental illness in England: Changes in equity of utilisation at the small area level between 2006 and 2010
Source: Soc Sci Med. 2014 Nov;120:243–51. doi: 10.1016/j.socscimed.2014.09.036 (PMC4225455; doi:10.1016/j.socscimed.2014.09.036)
Supplement: Supplementary file 1 [file mmc1.docx]

**Online appendix: Factors found to be associated with hospital admission for severe mental illness**

In section 1.2, the manuscript summarises the results of a literature review looking at which factors have been found to be associated with hospital admission for severe mental illness. The table below provides further detail and a full list of references, which have not been included in the manuscript for reasons of space.

| **Factor associated with hospital admission for SMI** | **Relevant articles** | **Notes on the direction and nature of the relationship** |
| --- | --- | --- |
| Age | [1] [2] [3] [4] [5] [6] | Higher for younger ages (Germany; Canada; US; Switzerland; Spain); not stated (Germany). |
| Alcohol abuse | [7] [8] [9] [10] [11] | Positively related (US veterans; US; US; US; US). |
| Drug abuse | [7] [12] [13] [14] [10] [15] [16] [17] [18] [19] [11] | Positively related (US veterans; Germany/Greece/Italy/Spain; Spain; US; US; US; US; US; Australia; Spain; US). |
| Education level | [20] [21] [4] [22] [23] | Negatively related (Sweden; Japan; Switzerland); positively related (US; US). |
| Ethnicity | [24] [25] [26] [27] [28] | Differing rates of compulsory admission (UK); higher schizophrenia rates and lower bipolar rates for black patients (US military); black patients more likely to be compulsorily detained (UK); black patients more likely to be admitted and more likely to be detained (UK); black patients more likely to be admitted (US). |
| Hospitalisation (number of previous SMI admissions) | [29] [13] [30] [31] [3] [14] [22] | Positively related (France; Spain; Canada; US; US; US; US). |
| Living alone | [29] [4] | Negatively related (France); positively related (Switzerland) |
| Medication non-adherence | [13] [20] [32] [33] [15] [16] [34] [19] [11] [35] | Positively related (Spain; Sweden; US; US; US; US; Spain; US; US; US). |
| Medication type | [15] [16] [36] [14] [10] [37] [38] [39] [40] [10] [41] [42] [43] | Various differences in hospitalisation between different drugs. |
| Rural area | [44] [45] [46] | Negatively related (US); positively related (Australia); negatively related (Germany). |
| Seasonality | [47] [48] [49] [50] [51] [52] [53] | Various effects for different diagnoses. |
| Severity / global severity | [1] [31] [12] [10] | Positively related (Germany; US; Germany/Greece/Italy/Spain; US). |
| Sex | [54] [13] [55] [56] [25] [6] | Higher age of first admission for men (Ireland); higher risk for young men (Spain); higher compulsory admission for men (France); higher admission rate for men (US); higher for women (US military); not stated (Germany). |
| Unemployment | [46] [44] [6] [23] | Positively related (Germany; US); not stated (Germany); positively related (US). |

References:

1. Ujeyl, M., et al., *Hospitalization in patients with schizophrenia - Which factors predict hospital readmission?* European Archives of Psychiatry and Clinical Neuroscience, 2009. **259**: p. S57-S58.

2. Toma, F.C. and J. Moamai, *Readmission risk in Schizophrenia and related disorders: Involuntary first hospitalization as a predictor.* Schizophrenia Research, 2010. **117 (2-3)**: p. 281.

3. Perlick, D.A., et al., *Symptoms predicting inpatient service use among patients with bipolar affective disorder.* Psychiatric Services, 1999. **50**(6): p. 806-812.

4. Lay, B., C. Lauber, and W. Rossler, *Prediction of in-patient use in first-admitted patients with psychosis.* European Psychiatry: the Journal of the Association of European Psychiatrists, 2006. **21**(6): p. 401-9.

5. Laura, V., et al., *Rates and predictors of re-hospitalization after the first lifetime hospitalizations for manic or mixed episode. A naturalistic study in Spanish sample of bipolar i inpatients.* International Clinical Psychopharmacology, 2012. **28**: p. e64-e65.

6. Doering, S., et al., *Predictors of relapse and rehospitalization in schizophrenia and schizoaffective disorder.* Schizophrenia Bulletin, 1998. **24**(1): p. 87-98.

7. Hoblyn, J.C., et al., *Substance use disorders as risk factors for psychiatric hospitalization in bipolar disorder.* Psychiatric Services, 2009. **60**(1): p. 50-5.

8. Gerding, L.B., et al., *Alcohol dependence and hospitalization in schizophrenia.* Schizophrenia Research, 1999. **38**(1): p. 71-5.

9. Cawood, F.W. and J.J. Bartko, *The correlation of alcohol consumption with schizophrenia hospitalizations: 1934 to 2005.* Schizophrenia Research, 2009. **111**(1-3): p. 194-195.

10. Olfson, M., et al., *Determinants of psychiatric hospital admission in schizophrenia.* Value in Health, 2010. **13 (7)**: p. A445.

11. Haywood, T.W., et al., *Predicting the "revolving door" phenomenon among patients with schizophrenic, schizoaffective, and affective disorders.* American Journal of Psychiatry, 1995. **152**(6): p. 856-61.

12. Gorwood, P., *Factors associated with hospitalisation of patients with schizophrenia in four European countries.* European Psychiatry: the Journal of the Association of European Psychiatrists, 2011. **26**(4): p. 224-30.

13. De La Vega Sanchez, D.C., et al., *Factors determining admission to psychiatric hospitalization in psychotic patients.* European Psychiatry, 2010. **25**.

14. Olfson, M., et al., *Assessing clinical predictions of early rehospitalization in schizophrenia.* Journal of Nervous & Mental Disease, 1999. **187**(12): p. 721-9.

15. Lang, K., et al., *Medication adherence and hospitalization among patients with schizophrenia treated with antipsychotics.* Psychiatric Services, 2010. **61**(12): p. 1239-47.

16. Lang, K., et al., *Predictors of medication nonadherence and hospitalization in Medicaid patients with bipolar I disorder given long-acting or oral antipsychotics.* Journal of Medical Economics, 2011. **14**(2): p. 217-26.

17. Shaner, A., et al., *Disability income, cocaine use, and repeated hospitalization among schizophrenic cocaine abusers--a government-sponsored revolving door?* New England Journal of Medicine, 1995. **333**(12): p. 777-83.

18. Sara, G., et al., *Amphetamine availability and admissions for psychosis in New South Wales, 2001-2009.* Australian & New Zealand Journal of Psychiatry, 2011. **45**(4): p. 317-24.

19. San-Molina, L., et al., *Reasons for relapses in patients with schizophrenia admitted to acute units.* European Psychiatry, 2011. **26**.

20. Boden, R., et al., *Early non-adherence to medication and other risk factors for rehospitalization in schizophrenia and schizoaffective disorder.* Schizophrenia Research, 2011. **133**(1-3): p. 36-41.

21. Suzuki, Y., et al., *Associated factors of rehospitalization among schizophrenic patients.* Psychiatry and Clinical Neurosciences, 2003. **57**(6): p. 555-561.

22. Klinkenberg, W.D. and R.J. Calsyn, *Gender differences in the receipt of aftercare and psychiatric hospitalization among adults with severe mental illness.* Comprehensive Psychiatry, 1998. **39**(3): p. 137-42.

23. Ramsay, C.E., et al., *Rates and clinical correlates of school drop-out, unemployment, and prior incarceration among hospitalized patients with firstepisode psychosis: Prevalent barriers to recovery.* Early Intervention in Psychiatry, 2010. **4**: p. 73.

24. Davies, S., et al., *Ethnic differences in risk of compulsory psychiatric admission among representative cases of psychosis in London.* BMJ, 1996. **312**(7030): p. 533-7.

25. Herrell, R., et al., *First psychiatric hospitalizations in the US military: the National Collaborative Study of Early Psychosis and Suicide (NCSEPS).* Psychological Medicine, 2006. **36**(10): p. 1405-15.

26. Commander, M.J., et al., *Mental health care for Asian, black and white patients with non-affective psychoses: pathways to the psychiatric hospital, in-patient and after-care.* Social Psychiatry & Psychiatric Epidemiology, 1999. **34**(9): p. 484-91.

27. Tolmac, J. and M. Hodes, *Ethnic variation among adolescent psychiatric in-patients with psychotic disorders.* British Journal of Psychiatry, 2004. **184**: p. 428-31.

28. Rost, K., et al., *Potential disparities in the management of schizophrenia in the United States.* Psychiatric Services, 2011. **62**(6): p. 613-8.

29. Sarlon, E., et al., *Predictive factors for hospitalization in French schizophrenic patients.* Value in Health, 2009. **12 (7)**: p. A353.

30. Addington, D.E., et al., *Predictors of admission in first-episode psychosis: developing a risk adjustment model for service comparisons.* Psychiatric Services, 2010. **61**(5): p. 483-8.

31. Postrado, L.T. and A.F. Lehman, *Quality of life and clinical predictors of rehospitalization of persons with severe mental illness.* Psychiatric Services, 1995. **46**(11): p. 1161-1165.

32. Wong, B., et al., *Among patients with schizophrenia non-adherence to antipsychotic medications early-on results in more hospitalizations and greater healthcare costs.* Early Intervention in Psychiatry, 2012. **6**: p. 99.

33. Law, M.R., et al., *A longitudinal study of medication nonadherence and hospitalization risk in schizophrenia.* Journal of Clinical Psychiatry, 2008. **69**(1): p. 47-53.

34. Weiden, P.J., et al., *Partial compliance and risk of rehospitalization among California Medicaid patients with schizophrenia.* Psychiatric Services, 2004. **55**(8): p. 886-91.

35. Hassan, M., et al., *Hospitalizations among bipolar disorder patients before and after initiating lurasidone in a commercially insured population.* Value in Health, 2013. **16 (3)**: p. A68.

36. Jing, Y., et al., *Comparison of second-generation antipsychotic treatment on psychiatric hospitalization in Medicaid beneficiaries with bipolar disorder.* Journal of Medical Economics, 2011. **14**(6): p. 777-86.

37. Gianfrancesco, F., et al., *Hospitalisation risks in the treatment of schizophrenia in a Medicaid population: comparison of antipsychotic medications.* International Journal of Clinical Practice, 2006. **60**(11): p. 1419-24.

38. Gianfrancesco, F., K. Rajagopalan, and R.H. Wang, *Hospitalization risks in the treatment of schizophrenia: comparison of antipsychotic medications.* Journal of Clinical Psychopharmacology, 2006. **26**(4): p. 401-4.

39. Gianfrancesco, F., et al., *Hospitalization risks in the treatment of bipolar disorder: comparison of antipsychotic medications.* Bipolar Disorders, 2007. **9**(3): p. 252-61.

40. Olfson, M., et al., *Predicting psychiatric hospital admission among adults with schizophrenia.* Psychiatric Services, 2011. **62**(10): p. 1138-45.

41. Stephansson, O., et al., *Prescribed antipsychotic drugs and risk of re-hospitalization among incident patients with schizophrenia-related diagnosis.* Pharmacoepidemiology and Drug Safety, 2010. **19**: p. S47-S48.

42. Caceres, M.C., et al., *Increased use of second generation antipsychotic drugs in primary care: potential relevance for hospitalizations in schizophrenia patients.* European Journal of Clinical Pharmacology, 2008. **64**(1): p. 73-6.

43. Broder, M.S., et al., *Association between second-generation antipsychotic medication half-life and hospitalization in the community treatment of adult schizophrenia.* Journal of Medical Economics, 2012. **15**(1): p. 105-11.

44. Fortney, J.C., S. Xu, and F. Dong, *Community-level correlates of hospitalizations for persons with schizophrenia.* Psychiatric Services, 2009. **60**(6): p. 772-8.

45. Sara, G. and P. Burgess, *'Second admission psychosis': Predictors of readmission within two years of a first episode.* Australian and New Zealand Journal of Psychiatry, 2012. **46**: p. 54.

46. Losert, C., et al., *Area characteristics and admission rates of people with schizophrenia and affective disorders in a German rural catchment area.* Epidemiology & Psychiatric Science, 2012. **21**(4): p. 371-9.

47. Ramos, I.V., et al., *Seasonal variations in bipolar disorder admissions in Galiza.* European Neuropsychopharmacology, 2009. **19**: p. S455-S456.

48. Morken, G., S. Lilleeng, and O.M. Linaker, *Seasonal variation in suicides and in admissions to hospital for mania and depression.* Journal of Affective Disorders, 2002. **69**(1-3): p. 39-45.

49. Hallam, K.T., et al., *Seasonal influences on first-episode admission in affective and non-affective psychosis.* Acta Neuropsychiatrica, 2006. **18**(3-4): p. 154-161.

50. Davies, G., et al., *Seasonality of first admissions for schizophrenia in the Southern Hemisphere.* Schizophrenia Research, 2000. **41**(3): p. 457-62.

51. Daniels, B.A., et al., *Seasonal variation in hospital admission for bipolar disorder, depression and schizophrenia in Tasmania.* Acta Psychiatrica Scandinavica, 2000. **102**(1): p. 38-43.

52. Clarke, M., et al., *Seasonal influences on admissions in schizophrenia and affective disorder in Ireland.* Schizophrenia Research, 1998. **34**(3): p. 143-9.

53. Clarke, M., et al., *Seasonal influences on admissions for affective disorder and schizophrenia in Ireland: a comparison of first and readmissions.* European Psychiatry: the Journal of the Association of European Psychiatrists, 1999. **14**(5): p. 251-5.

54. Hart, C., D.T. Doherty, and D. Walsh, *First admissions to inpatient care in Ireland for schizophrenia 1971-2004: Trends in age and gender.* Irish Journal of Psychological Medicine, 2007. **24**(4): p. 132-139.

55. Cougnard, A., et al., *Factors influencing compulsory admission in first-admitted subjects with psychosis.* Social Psychiatry & Psychiatric Epidemiology, 2004. **39**(10): p. 804-9.

56. Kleinhaus, K., et al., *Age, sex and first treatment of schizophrenia in a population cohort.* Journal of Psychiatric Research, 2011. **45**(1): p. 136-141.
